# Reassessment of pre-industrial fire emissions strongly affects anthropogenic aerosol forcing

**DOI:** 10.1038/s41467-018-05592-9

**Published:** 2018-08-09

**Authors:** D. S. Hamilton, S. Hantson, C. E. Scott, J. O. Kaplan, K. J. Pringle, L. P. Nieradzik, A. Rap, G. A. Folberth, D. V. Spracklen, K. S. Carslaw

**Affiliations:** 1 School of Earth and Environment, University of Leeds, Leeds, LS2 9JT UK; 2 Department of Earth and Atmospheric Science, Cornell University, Ithaca, 14853 NY USA; 3 Karlsruhe Institute of Technology, Institute of Meteorology and Climate research, Atmospheric Environmental Research, 82467 Garmisch-Partenkirchen, Germany; 4 Geospatial Data Solutions Center, University of California Irvine, California, 92697 USA; 5 ARVE Research SARL, Pully, 1009 Switzerland; 6 Environmental Change Institute, School of Geography and the Environment, University of Oxford, Oxford, OX1 3QY UK; 7 Max Planck Institute for the Science of Human History, Jena, 07745 Germany; 8 Institute for Physical Geography and Ecosystem Sciences, Lund University, Lund, S-223 62 Sweden; 9 CSIRO Oceans and Atmosphere, Canberra, ACT 2601 Australia; 10 Met Office Hadley Centre, Exeter, EX1 3PB UK

## Abstract

Uncertainty in pre-industrial natural aerosol emissions is a major component of the overall uncertainty in the radiative forcing of climate. Improved characterisation of natural emissions and their radiative effects can therefore increase the accuracy of global climate model projections. Here we show that revised assumptions about pre-industrial fire activity result in significantly increased aerosol concentrations in the pre-industrial atmosphere. Revised global model simulations predict a 35% reduction in the calculated global mean cloud albedo forcing over the Industrial Era (1750–2000 CE) compared to estimates using emissions data from the Sixth Coupled Model Intercomparison Project. An estimated upper limit to pre-industrial fire emissions results in a much greater (91%) reduction in forcing. When compared to 26 other uncertain parameters or inputs in our model, pre-industrial fire emissions are by far the single largest source of uncertainty in pre-industrial aerosol concentrations, and hence in our understanding of the magnitude of the historical radiative forcing due to anthropogenic aerosol emissions.

## Abstract (web summary)

Several lines of evidence suggest that fire activity was much greater in the preindustrial era than currently assumed in climate models. Here the authors show that greater emission of aerosols from fires leads to a substantial reduction in the magnitude of aerosol radiative forcing over the Industrial Era.

## Introduction

**Fig. 1:**
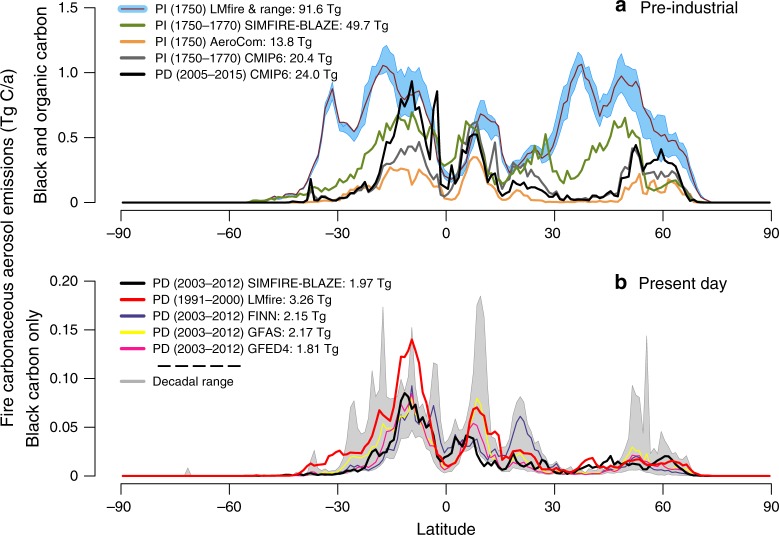
Fire carbonaceous aerosol emissions. **a** Pre-industrial (PI) and present-day (PD) carbonaceous fire emissions (sum of black and organic carbon) as a function of latitude. PI fire emissions derived from four fire datasets: LMfire, SIMFIRE-BLAZE, CMIP6 and AeroCom. PD fire emissions are from the CMIP6 dataset. The range around LMfire represents a plausible range in the natural variability of PI fire emissions derived from the maximum and minimum in fire emissions from the four distinct decadal mean fire climatologies. Seasonal PI carbonaceous aerosol emission maps are shown in Supplementary Figure 4. **b** Black carbon emissions for PD from five datasets: LMfire, SIMFIRE-BLAZE, GFED4, GFAS and FINN (mean of 2003–2012 for all except LMfire which is the mean over 1991–2000). Shaded area represents the minimum and maximum in decadal emissions from within all three observation datasets

The global occurrence of wildfire and biomass burning in the modern world is controlled by a combination of climate and human activity^1–3^. The present-day (PD) pattern of fire (Fig. 1) is relatively well understood based on global satellite measurements of burned area^4^. However, despite the importance of fire in the climate system in the past and present^5^, current understanding of fire occurrence in the late pre-industrial Holocene (PI) is limited^6^. Analyses of ice core records, charcoal measured in lake and marine sediments, and tree-rings suggest that fire activity varied considerably over the last 500 years, but that generally fire occurrence increased to a peak around 1850 CE before declining to PD levels^7–13^. Paleoenvironmental archives therefore suggest that PI fire activity was similar to PD activity, if not higher (Supplementary Figures 1 and 2 and Supplementary Table 1). This runs contrary to existing ideas about the pristine nature of the Earth system in the PI, which is embodied in both the AeroCom 1750^14^ and the Coupled Model Intercomparison Project (CMIP) phase 5 and 6 aerosol emission datasets^15,16^.

It has been widely assumed in global climate models that aerosol emissions from fires in the PI were lower than in the PD^14–16^, based on a misconception that total fire emissions have increased with human population density^17^. Globally, most fire ignitions are caused by humans^1^, which makes a positive scaling of total burned area, and hence total fire emissions, with human population density logical at first. However, recent analysis of global fire occurrence shows that, at a global scale, burned area declines with increasing population density^18,19^ due to land use change^20^. For example, observationally based estimates of burned area over the last few decades suggest a global decline of 1–2% per year^20–22^, with a maximum of 3–6% per year regional decline in Europe and Australia/New Zealand^20,21^. On longer timescales Mallek et al.^23^ suggest that PD (1984–2009) burned area is just 14% of PI (1500–1850) burned area in California, and Arora and Melton^24^ suggest an overall global decline of 25–30% in burned area since the PI. This decline in fire is a result of human activity: e.g., passive fire suppression from landscape fragmentation limits the spread of fires^25^, while active fire suppression management and legislation aimed to improve air quality offset any potential anthropogenic increase in accidental fire ignitions^26^. Therefore, advances in global fire modelling^27^ suggest a significantly different pattern of fire occurrence under PI conditions (modelled PI burned area estimates: Supplementary Figure 3), with consequently higher aerosol emissions than previously estimated.

A large number of global fire models exist, of which six contributed to the creation of historical fire emissions in the CMIP6 dataset^16^. However, most fire models still assume an overall positive relationship between human population and fire^27^ so they do not capture the observed human-driven decline in burned area, and hence fire emissions, over the last decades^20^. Therefore, CMIP6 probably underestimates PI fire emissions.

Here we use two fire models driven by distinct philosophies of the relationship between humans and fire to simulate a range of PI fire emissions which are consistent with current knowledge of how humans influence global fire occurrence, as well as representing the uncertainty in anthropogenic land cover and land use change from the PI to the PD (see Methods). Emissions in the two fire models are much greater than in the AeroCom and CMIP6 PI datasets and are more in line with the trends predicted from paleofire proxy records. When used in a global aerosol model, the fire models result in a substantial reduction in the magnitude of anthropogenic aerosol forcing over the Industrial Era compared to the CMIP6 fire emission scenario; which has not been accounted for in climate models.

## Results

### Modelling pre-industrial fire emissions

To quantify how assumptions concerning PI fire emissions affect historical aerosol concentrations, and how this change in the PI aerosol baseline affects anthropogenic radiative forcing, we use four datasets of black carbon (BC), particulate organic matter (POM) and sulphur dioxide (SO_2_) fire emissions in a global aerosol model (Supplementary Table 2). We define the PI to be 1750 and fire emissions are defined as the sum of emissions from natural wildfires as well as fires resulting from anthropogenic activity.

The principal fire modelling dataset is calculated using the LPJ-GUESS-SIMFIRE-BLAZE model. To calculate burned area BLAZE incorporates the empirically optimised SIMFIRE (SIMple FIRE) model^18,28^. SIMFIRE-BLAZE is then configured to run under PI conditions, but does not explicitly simulate PI agricultural burning. We also calculate PI fire emissions using the LPJ-LMfire^29^ model. LMfire is currently the only fire model specifically designed to simulate human-caused fires and natural wildfires in the PI period. LMfire reproduces long-term observed PD boreal fire behaviour within central Alaska^29^, which is the PD region where fire activity is most analogous to the PI^30^. We compare PI fire emissions from these two fire models with emissions from the CMIP6 dataset^16^, which is the most recent fire emission dataset for use within global climate and Earth system models. The CMIP6 dataset for both the PI and PD combines information from satellite measurements, fire emission proxy records and six fire models from the Fire Model Intercomparison Project (FireMIP; see Methods). To assess how the CMIP6 fire dataset and our new fire models compare to earlier understanding of PI fire emissions we also compare to the Dentener et al.^14^ 1750 dataset adopted by AeroCom. A global aerosol model was then used to quantify the changes in PI aerosol concentrations resulting from each fire emission dataset. We also calculate the effect on the aerosol radiative forcing between the PI and PD assuming a common observation-based fire emission dataset for the PD, which is also used in CMIP6 (see Methods).

### Evaluation of fire emissions

The four PI fire emission datasets (AeroCom, CMIP6, SIMFIRE-BLAZE and LMfire) produce a wide range of aerosol and trace gas emissions (Supplementary Table 2). Figure 1 shows that when assuming a positive relationship between human population and fire occurrence (AeroCom scenario), fire emissions are consistently lower in the PI than the PD, because emissions are scaled only by population changes in most regions. Global annual mean PI CMIP6 carbonaceous fire emissions are 48% higher than in the AeroCom dataset, with major increases over Northern Hemisphere (NH) mid-latitudes and tropical Africa, but are still lower than the PD. In contrast, although the LMfire and SIMFIRE-BLAZE modelled PI fire emissions differ substantially, they are both much higher than PD emissions at almost all latitudes outside the tropics, where continental pristine aerosol environments are both spatially and temporally rare^30^. The majority of PD fire emissions originate in tropical savannah and grassland regions^31^ where herbaceous biomass accumulates during the wet season and provides large quantities of finely structured fuel that can readily burn during the dry season. In the PI a significant fraction of global emissions also originate from NH mid-latitudes, while in the PD passive and active human fire suppression is highly active (e.g., >99.5% fire ignitions are actively suppressed in the US^32^). Different representations of anthropogenic land cover and land use change over the industrial period between the two fire models (see Methods) contribute to the significant differences in modelled estimates of PI burned area (Supplementary Figure 3) and emissions (Supplementary Figure 4); particularly between 30° N and 45° N where agricultural emissions present in LMfire but not SIMFIRE-BLAZE are estimated to contribute up to 25% (Eurasia: 37%, North America: 1%) of total fire emissions, and during the boreal spring season. Recent studies suggest that mid-latitude Eurasian post-harvest agricultural burning emissions have been strongly underestimated in both the PI^33^ and PD^34,35^, so the inclusion of these sources in LMfire, but not SIMFIRE-BLAZE, adds significantly to the total emissions between 30° N and 45° N, but further research is needed to determine their accuracy.

Depending on the emitted species, modelled total global fire emissions in the PI are estimated to be between approximately two-and-a-half and five times higher than those in the CMIP6 dataset (Supplementary Table 2), reflecting the large contribution to the uncertainty in fire emissions from fire modelling processes and assumptions about land use change (see Methods). These differences also lie well outside the perturbations assumed in multi-model sensitivity studies^36^, and have a different spatial distribution due to differences in fire emissions corresponding with changes in the location of PI fire occurrence rather than a uniform global increase. Seasonal patterns in fire emissions are similar between fire models, except in spring where LMfire simulates significantly more emissions than SIMFIRE-BLAZE in both hemispheres (Supplementary Figure 4). Overall, the fire model simulations suggest that a large source of PI aerosol emissions is currently missing from climate models and the CMIP6 experiments.

Figure 1 also compares SIMFIRE-BLAZE and LMfire modelled BC emissions for the PD climate (see Methods) along with the range in BC emissions derived from all three commonly used fire inventories (The Global Fire Emissions Database: GFED4, The Global Fire Assimilation System: GFAS and the Fire INventory from NCAR: FINN). SIMFIRE-BLAZE and LMfire emissions generally lie within the decadal-mean emission range of the three observation-based datasets, although LMfire emissions are consistently higher in SH regions compared to decadal mean values from all other datasets. We also note that the two observation-based inventories can differ by up to a factor 3 themselves, and therefore a factor 2 difference at mid-latitudes between SIMFIRE-BLAZE and LMfire emissions in the PI reflects the uncertainty which also hinders accurate estimates of PD emissions.

The ability of a model to reproduce PD emissions does not necessarily mean that it also produces realistic results under a different past climate using different boundary conditions and where emissions are controlled by different processes. Hence, we advocate that model performance is best evaluated against what we know about the PI, rather than the PD.

### Comparisons with ice core records

**Fig. 2:**
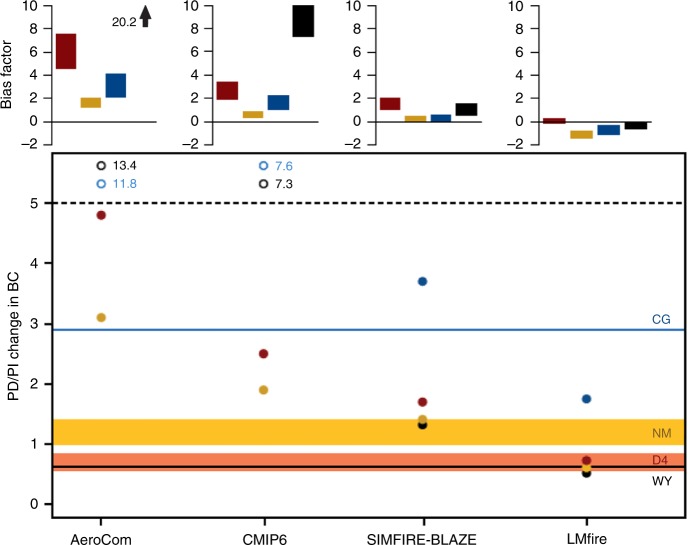
Present-day/pre-industrial ratio in ice core and modelled black carbon concentrations. Shown are two Greenland sites (D4 and NM), one North American site at Wyoming (WY), and one Swiss Alps site at Colle Gnifetti (CG). Bias factor in the present-day/pre-industrial (PD/PI) ratio for each modelled fire emission scenario (D4: red bars, NM: gold bars, WY: black bars, CG: blue bars). For model M and observation O the symmetric bias factor was calculated as: For M > O: (M/O)–1; For O > M: –(O/M) + 1. The AeroCom bias factor at the WY site is off the scale and shown as an arrow with its bias factor given. Measured PD/PI values shown as horizontal bars (±standard error of the mean bound Greenland estimates, see Methods) with the model simulations PD/PI ratio shown as dots (D4: red, NM: gold, WY: black, CG: blue). The WY and CG modelled ratio changes are scaled by a factor of 1.6 (1.2–2.0 in bias factor panel) and 2.0 (1.5–2.5 in bias factor panel) respectively to account for the change in BC concentration emissions near the site between the period of the PD measurement and the modelled concentration (see Methods). The AeroCom and CMIP6 changes at the WY and CG sites are off the scale and shown as open circles with the PD/PI ratio given

BC deposited on glaciers and recovered in ice cores can be used to infer the relative change in atmospheric total BC concentrations that occurred between the PI and the PD^11^. Here we evaluate NH changes in BC concentrations in each dataset. We compare modelled PD/PI BC atmospheric concentration ratios (see Methods, Supplementary Figure 5) to measured BC ice core ratios at four ice core locations: two in Greenland, and one in each of North America and Europe (Fig. 2). To minimise the large uncertainties associated with the absolute deposition^37^, we analysed only the ratio of PI to PD BC in the cores, assuming that trends can be more accurately modelled than absolute values. Our hypothesis is that a modelled PD/PI ratio that is higher than measured indicates that PI emissions are too low in the model compared to the PD. Although absolute BC deposition is uncertain^37^ (see later discussion) we assume that the controlling factor in the PD/PI ratio across multiple independent sites will be the trend in emissions. Together these ice core records provide an estimate of how fire emissions have changed within, or downwind of, NH regions of intensive land use and land cover change over the last 200 years^38,39^.

Overall (Fig. 2), global aerosol model simulations of the PD/PI ratio in BC concentrations using LMfire and SIMFIRE-BLAZE fire emissions are in closer agreement with ice core measurements than the global aerosol model simulation which uses the CMIP6 fire emissions dataset, although CMIP6 is an important improvement on the AeroCom dataset.

In Greenland the ice core BC in the PI is derived from wildland fires^10^ transported from boreal North America^40^, while in the PD it will also contain large amounts of BC from industrial activity^41^. Figure 2 compares the aerosol model with the PD/PI ratio in ice core BC concentrations from the D4 core (2713 m above sea level) in central Greenland^10^ and the NEEM core (2480 m above sea level) in northern Greenland^42^. Using AeroCom emissions in the aerosol model results in a PD/PI atmospheric BC ratio that is a factor 2.2–3.1 higher at NEEM and a factor 5.6–8.6 higher at D4 than the measured BC ratio in the ice. These ratios suggest that assuming a positive relationship between human population density and fire occurrence results in PI fire emissions that are too low. Using CMIP6 emissions results in a PD/PI BC ratio that is a factor of 1.3–1.9 higher at NEEM and a factor of 2.9–4.5 higher at D4 than the measured BC ratio. Using LMfire emissions results in a PD/PI BC ratio that is a factor 1.7–2.4 lower at NEEM and between a factor of 1.3 higher and a factor of 1.1 lower at D4, while using SIMFIRE-BLAZE emissions results in a PD/PI BC ratio that is between a factor of 1.0–1.5 higher at NEEM and a factor of 2.0–3.1 higher at D4, than the measured BC ratio.

In the Wyoming ice core^12^, which is 4100 m above sea level (see Methods), measured PD (1970–1979) BC concentrations are 37% lower than in the PI (Fig. 2). However, North American industrial BC emissions have been decreasing significantly since 1970^41,43^, so to compare with our model simulation in 2000 we scale the model PD concentrations by a factor of 1.6 ± 0.4 in line with the CMIP6 changes in anthropogenic emissions, and the uncertainty, over this period (see Methods). The global aerosol model with AeroCom fire emissions results in a PD/PI BC ratio at the altitude of the measurement site that is a factor 21.2 (15.7–26.2) larger than the measured ratio in the ice core (0.6), again consistent with the hypothesis of fire emissions being too low in the PI with a positive scaling of fire occurrence with population density. Using CMIP6 emissions results in a PD/PI ratio that is a factor of 11.5 (8.5–14.2) higher than the measured BC ratio. Using LMfire emissions results in a PD/PI BC ratio that is a factor 1.2 (1.0–1.6) lower than measured, while using SIMFIRE-BLAZE emissions results in a PD/PI BC ratio that is 2.2 (1.6–2.7) higher than measured. Aerosol model results using fire model estimates of PI fire emissions are much closer to the measured PI to PD change in BC concentrations than the aerosol model results using the AeroCom and CMIP6 datasets, which predict far larger increases in BC from PI to PD.

In the Colle Gnifetti ice core in the Swiss Alps, which is 4450 m above sea level^44^ (see Methods), BC concentrations averaged for 1950-to-1980 are a factor of 2.9 higher than in 1750 (Fig. 2). Total BC concentrations at this site are significantly higher in the PD than in the PI due to additional industrial emissions that are not present in the PI. Similarly to North America, European industrial BC emissions have been decreasing significantly since 1960^45^. In Fig. 2 we account for this known change by increasing the global aerosol model PD concentrations of BC by a factor of 2.0 ± 0.5 (see Methods). The global aerosol model with AeroCom fire emissions results in a PD/PI BC ratio, at the altitude of the measurement site, that is a factor 4.1 (3.1–5.2) larger than the PD/PI BC ratio recorded in the ice core; again, consistent with AeroCom PI emissions being too low. Using CMIP6 emissions results in a PD/PI ratio that is a factor of 2.7 (2.0–3.3) higher. Using LMfire emissions results in a PD/PI BC ratio that is a factor 1.6 (1.3–2.2) lower than the ratio recorded in the ice core, while using SIMFIRE-BLAZE emissions results in a PD/PI BC ratio that is 1.3 (1.0–1.6) higher than the ratio recorded in the ice core.

Due to the short atmospheric lifetimes of aerosol and soluble gases, ice cores store local-to-hemispheric-scale changes and contain many uncertainties as a proxy for fire emissions (see Methods). Nevertheless, we find that the changes in BC recorded in ice cores at four sites in very different environments are inconsistent with the assumption that fire emissions were significantly lower in the PI than in the PD, especially over North America and Europe. Compared to using fire emissions from the CMIP6 dataset, incorporating fire model emissions within the aerosol model results in closer agreement with the measured PD/PI ratio of total BC.

### Comparisons with other paleofire proxy records

Other proxies for fire occurrence and emissions provide further evidence for the likely changes in fire occurrence from the PI to the PD. Arora and Melton^24^ recently reported a 28% decline in global burned area from the PI (1850–1870) to PD (2005–2010). Applying this change to PD estimates of burned area from GFED4s (~475 Mha)^24^ and FINN (~725 Mha)^46^ results in an estimated PI range in burned area from 679 to 1040 Mha (Supplementary Table 3). Emissions from SIMFIRE-BLAZE in the PI are based on a burned area of 641 Mha, while emissions from LMfire are based on a burned area of 1180 Mha (Supplementary Figure 3). This would suggest that burned area in SIMFIRE-BLAZE is below the lower end of the range in PI burned area and LMfire is above the upper end of the range. However, Arora and Melton state that their burned area estimates do not include contributions from agricultural burning, which are a significant proportion of emissions at mid-latitudes in LMfire, suggesting that LMfire represents the plausible upper limit of PI emissions in many regions. A regional comparison of modelled burned area with the Arora and Melton dataset^24^ suggests that the distribution of burned area, and hence emissions, in LMfire is appropriate, although the magnitude of the emissions is high.

The charcoal record (see Supplementary Figure 2) suggests that fire occurrence in the tropics (30° S to 30° N) increased slightly from the PI to the PD. Moreover, in general agreement with Fig. 1, fire occurrence in the extra-tropical regions (>30° S and N) in the PI was similar to or larger than in the year 2000^8,9^. The charcoal record suggests fire occurrence in the NH mid-latitude region (25–45° N) is lower in the PD compared to the PI, due mainly to a decrease in charcoal deposition occurring over the last few decades, the same region in which the fire models (Fig. 1) predict the largest decrease in PD emissions compared to the PI, and where tree-ring-based reconstructions of fire activity for western North America also suggests a very rapid decline in fire occurrence from the PI to the PD^13^.

Greenland ice core measurements of unique chemical tracers of fires are also consistent with a decrease in fire emissions since the PI (see Supplementary Figure 1 and Supplementary Table 1). We note that CO measurements in Antarctic ice cores^7^ also suggest that PI fire emissions are higher over the Southern Hemisphere than are currently estimated in inventories. Although the measured quadrupling of the enhancement in PI CO from fires cannot be reproduced from PD fire distributions^6^, a significant change in fire emissions from nearby regions such as Patagonia and Australia (such as shown within the fire modelling presented here) could significantly enhance PI CO from fires transported to Antarctica.

Overall, the CMIP6 fire emission inventory does not capture the trends in these proxies, which are more closely in agreement with the overall trends in fire occurrence calculated from fire modelling, which specifically incorporates information on the non-linear relationship between fire occurrence and human population density. In terms of fire model performance, neither model outperforms the other in all regions and each has its own strengths (see Methods). Based on currently available measurements and the limited comparison with ice core records, we suggest that LMfire represents what is likely to be an upper limit to the plausible range of PI fire emissions in many regions, particularly in the extra-tropics.

### Pre-industrial aerosol concentrations

**Fig. 3:**
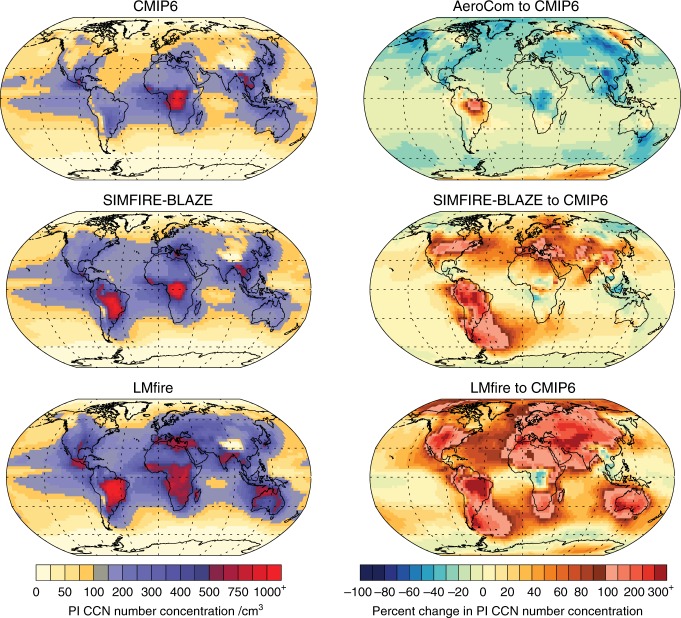
Pre-industrial cloud condensation nuclei concentrations. Annual mean pre-industrial cloud condensation nuclei (CCN) concentrations (cm^−3^) for the three main PI fire emission datasets (LMfire, SIMFIRE-BLAZE and CMIP6), and the percent change in the AeroCom, SIMFIRE-BLAZE and LMfire fire emission estimates compared to the CMIP6 dataset. CCN number concentrations are calculated at a supersaturation of 0.2% at 915 hPa (approximately cloud base for warm shallow, radiatively important clouds)

Figure 3 shows annual mean PI cloud condensation nuclei (CCN) concentrations simulated by the aerosol model for the four fire emission scenarios (see Methods). Global monthly mean PI CCN concentrations at 915 hPa (approximately low-level warm cloud base) are 110 cm^−3^ when using CMIP6 emissions; this is a factor of 1.1 higher than those calculated using AeroCom emissions. Compared to using CMIP6 emissions, CCN concentrations are a factor of 1.3 higher when using SIMFIRE-BLAZE emissions and a factor 1.7 higher when using LMfire emissions (see Supplementary Figure 6). Over continental regions, the increases in CCN concentrations are even greater relative to using the CMIP6 emissions, a factor of 1.5 higher using SIMFIRE-BLAZE emissions and a factor of 2.1 higher using LMfire emissions. A large portion of this difference is due to substantially increased fire emissions in the NH mid-latitudes (Fig. 1). In general, using CMIP6 emissions results in increases in CCN concentrations in all regions compared to using AeroCom emissions, the exceptions being near the high PD deforestation regions of Brazil and both polar regions. Over Africa, CCN concentrations are higher in extra-tropical regions but lower in tropical regions when incorporating LMfire or SIMFIRE-BLAZE fire emissions compared to CMIP6. Over Indonesia and northern Australia CCN concentrations are higher when incorporating LMfire emissions compared to using CMIP6 emissions, while using SIMFIRE-BLAZE emissions yields lower CCN concentrations. Downwind of southern Australia and Patagonia both fire model emission datasets lead to higher CCN concentrations than when using CMIP6 fire emissions, but similar or lower CCN concentrations are calculated over most of the Southern Ocean when using the two fire models. Incorporating SIMFIRE-BLAZE and LMfire emissions results in marine CCN concentrations that are 1.2–1.5 times higher than when using CMIP6 emissions, due to long-range aerosol transport. The current study shows that pre-industrial fire emissions cause larger uncertainty in CCN (and hence pre-industrial to present-day aerosol indirect forcing) than the combined estimate in Hamilton et al.^30^ from 28 uncertain parameters related to aerosol processes and emissions.

### Effect on PI to PD anthropogenic aerosol radiative forcing

**Fig. 4:**
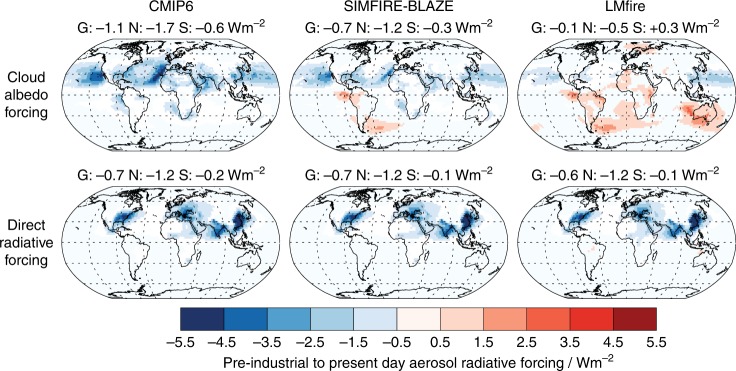
Annual mean pre-industrial to present-day aerosol cloud albedo forcing and direct radiative forcing. Annual mean radiative forcing values are given above each map for Global (G), Northern Hemisphere (N) and Southern Hemisphere (S) regions

Changes in PI CCN concentrations alter both the size and number of cloud drops in the PI, and therefore the magnitude of the cloud radiative perturbation caused by anthropogenic emissions over the historical period^47^. Figure 4 shows the radiative forcing due to changes in cloud albedo between the PI and PD using PI fire emissions from the CMIP6 dataset or the fire model datasets (see Methods). Other cloud adjustments caused by the changes in aerosol were not included, but are likely to enhance the albedo effect we calculate^43^. The global annual mean cloud albedo forcing in the simulation with CMIP6 emissions is −1.1 W m^−2^ in this model, a decrease of 16% compared to using the AeroCom PI and PD fire emission datasets (see Methods and Supplementary Table 2), but is reduced to −0.73 W m^−2^ in the SIMFIRE-BLAZE simulation and to −0.10 W m^−2^ in the LMfire simulation. All simulations show a similar latitudinal distribution in the forcing (Supplementary Figure 7), driven primarily by NH anthropogenic industrial emissions. We estimate the LMfire cloud albedo forcing to lie between −0.06 and −0.17 W m^−2^, based on four emission scenarios representing the mean decadal maximum and minimum in tropical and extra-tropical fire emissions simulated by LMfire (see Methods, Supplementary Table 2 and Supplementary Figure 8). This suggests that in order to improve the accuracy of radiative forcing estimates in climate models, it is more important to reduce the structural uncertainty between fire models than to improve our understanding of natural variability in PI fire emissions.

Figure 4 also shows the aerosol direct radiative forcing between the PI and PD for each fire emission scenario. Each PI fire emission experiment results in a similar spatial pattern of direct forcing. The global mean differences are much smaller than for the cloud albedo forcing, ranging from −0.68 W m^−2^ for CMIP6 PI fire emissions to −0.62 W m^−2^ for LMfire PI fire emissions (Supplementary Table 2). The sensitivity of the direct radiative forcing to PI fire emissions is weaker than for the cloud albedo forcing. This is caused by (i) the more linear response of the natural aerosol direct radiative effect compared to its cloud albedo effect^48^ and also by (ii) the smaller direct radiative efficiency of fire aerosol compared to other natural aerosol^48^ due to contrasting optical properties of absorbing BC and scattering POM.

The revised assumptions about PI fire emissions lead to substantial changes in the regional magnitude and pattern of the net aerosol radiative forcing over the industrial period. Our modelling results imply a substantial change in our understanding of the way the energy balance of the atmosphere has evolved over the industrial period, particularly over the Atlantic, with several possible implications for climate studies. In particular, compared to the simulation using CMIP6 fire emissions, we estimate that the revised fire emissions reduce the magnitude of the net (cloud albedo and direct) aerosol radiative forcing contrast between the Northern and Southern Hemispheres by 0.2 W m^−2^ for both the SIMFIRE-BLAZE and LMfire simulations, but with very different spatial distributions (Fig. 4). In many coupled atmosphere-ocean climate models, the inter-tropical convergence zone would likely be positioned further southward in the PI compared to models participating within CMIP6, as tropical precipitation patterns are sensitive to changes in the gradient in the inter-hemispheric aerosol forcing^49,50^. Other dynamical features of the climate system, such as Atlantic and Pacific storm tracks are potentially also sensitive to NH aerosol forcing^51,52^.

The inclusion of more realistic PI fire emissions in climate and Earth system models is likely to cause a general reduction in the magnitude of the aerosol radiative forcings that they simulate^53^, although limitations to cloud droplet concentrations that are imposed in some models will influence how they respond^54^. Any subsequent adjustment to climate model processes through tuning, while still maintaining agreement with historical global mean temperature changes, will affect the climate sensitivity of the models, and hence future climate projections^55,56^.

A realistic lower bound for PI fire emissions is currently unknown, but analysis of ice cores, charcoal records and tree-rings suggests it will be nearer to SIMFIRE-BLAZE modelled estimates than those within the AeroCom or CMIP6 datasets. LMfire emissions are approximately double SIMFIRE-BLAZE in most mid-latitude regions due to the addition of agricultural emissions and a longer fire season, which both increase annual mean burned area and hence modelled emission estimates. We therefore suggest that in many extra-tropical regions LMfire represents a plausible upper limit, particularly in the Arctic and semiarid regions of the mid-latitude NH. The exception is temperate North America, a region important for the baseline forcing, where reconstructions based on paleoenvironmental archives are in closest agreement with LMfire of all examined datasets. However, LMfire emissions from Australia are close to an order of magnitude higher than SIMFIRE-BLAZE and should be treated cautiously in the absence of supporting evidence, in particular the impact that colonisation has had on fire activity within Australia which could be large. The upper limit of potential fire activity in most tropical regions is also currently unknown.

### Uncertainties and future directions

Several lines of evidence, aside from more realistic fire models, point to PI fire emissions being greater than previously assumed: ice core records of the PI to PD change in BC at several independent sites in different environments; ice-core records of tracers of fire activity; recent analyses of fire occurrence revealing declining burned area with increasing population density (e.g., refs. ^13,18–20,24^); and indirect evidence from the charcoal record and tree-rings. Nevertheless, evidence to support new model estimates currently contains many uncertainties, including: the modelling of aerosol deposition on snow, in particular the extent to which models can simulate trends even if absolute deposition is very uncertain; the ability to simulate deposition to high-altitude sites using low-resolution models; the extent to which deposition scales with emissions and concentrations on a long-term mean; the effect of PI to PD changes in aerosol processing, atmospheric circulation and precipitation patterns; uncertainties in the different records of fire emissions themselves; uncertainty in land cover and land use change over the industrial period, and how this translates to uncertainty in aerosol emission and deposition rates; uncertainty in the impacts of European colonisation on fire regimes influenced by aboriginal land use practices; uncertainty in fire emission factors, which also may have been different in the PI environment; spatial and temporal variability in fire emissions, including the fire return interval, and how this affects the optimum length of model simulations; and the uncertainty in a single model analysis. These are clear tasks for future research to help reduce uncertainty in PI to PD changes in fire emissions, as well as the associated uncertainty in radiative forcing estimates over the Industrial Era, noting that any future constraints are not automatically equivalent within each time-period.

Our results highlight the importance of developing a much deeper understanding of how fires evolved over the industrial period, especially since global fire models have mostly been evaluated against PD data^27^. The exponential increase in human population over the Industrial Era has restructured the fire landscape on all inhabited continents and created a permanent change in the energy balance of the atmosphere that has not been fully accounted for in climate models. Furthermore, it is highly likely that models participating in CMIP6 would simulate smaller anthropogenic aerosol radiative forcings over the industrial period if they used higher PI fire emissions.

The climate impacts of fires will also extend beyond changes in aerosol emissions, and includes many other important processes^5^ such as changes in CO_2_ exchange, surface albedo, ozone and methane concentrations, as well as other smoke-induced adjustments to clouds and regional meteorology that were not explored here. These factors could amplify or attenuate the effects we have simulated here. A reduction in the uncertainties in unobservable PI aerosol concentrations^57–59^, including but not limited to fire emissions investigated within this study, will best be achieved through a multi-disciplinary approach, including contributions from Earth system modelling, palaeontology, geology, anthropology and archaeology.

## Methods

### AeroCom fire emissions

The AeroCom inventory consists of multiple datasets of global natural and anthropogenic aerosol and precursor emissions for the pre-industrial (PI) year 1750 and the present day (PD) year 2000^14^. AeroCom PI fire emissions are calculated by scaling back present day (year 2000) fire emissions from the Global Fire Emissions Database (GFED2)^60^ database according to the change in human population density between the PI and PD. Deforestation fires are scaled solely by population density data while fire emissions from all other land surface types are scaled to 60% of their PD value, assuming 40% would burn naturally. Exceptions for high latitude boreal regions were made to account for changes in fire suppression and are assumed to be double PD levels.

### Coupled Model Intercomparison Project phase 6 fire emissions

The Coupled Model Intercomparison Project (CMIP) phase 6 fire emission inventory^16^ provides monthly fire emissions from 1750 to 2015 anchored to GFED4s PD (1997–2015) emissions. Estimates of historical fire emissions are derived by merging the satellite record with fire proxy records (fire tracers contained within snow and ice and the charcoal record) in regions with suitable proxy coverage (North America and Europe), and by using the mean of six fire models in regions of limited proxy record coverage. Within the tropical rainforest regions of Equatorial Asia (including Indonesia, Micronesia, and Polynesia) and South America fire emissions are held constant back in time from 1960 and 1970, respectively. Agricultural fires are also kept relatively constant over time. Here we use the mean of emissions from 1750 to 1770 to represent the PI and the mean of emissions from 2006 to 2015 to represent the PD. The CMIP6 PI dataset is presented here as a lower bound to, and likely underestimates, the magnitude of PI fire emissions because the fire models used to construct the main part of the inventory are unable to reproduce the observed 24% decrease in global burned area over the last 18 years due to human impact^20^.

### SIMFIRE-BLAZE fire model description

The principle fire modelling dataset is a transient simulation using the LPJ-GUESS-SIMFIRE-BLAZE model, where PI is defined as the mean of 1750-to-1770 and PD is defined as the mean of 2003-to-2012. The BLAZE (L.P.N. et al., manuscript in preparation) combustion model simulates fire driven fluxes within the biosphere and from biosphere to atmosphere. It computes survival-probabilities for stands of trees based on the vegetation properties and potential fire-line intensity (FLI)^61^, which is derived from available fuels and local meteorology. In the event of a fire the survival probability of each tree is tested individually. The total area burned is computed by SIMFIRE (SIMple FIREmodel)^28^ using an algorithm fitted to values of average annual maximum fraction of absorbed photosynthetically active radiation (fAPAR), the maximum annual Nesterov-index, the biome-type, and human population density. The reciprocal of the monthly fractional area of grassland burned is used as a stochastic proxy for the occurrence of fire affecting forested regions in each grid cell. Fluxes between live and litter pools as well as the atmosphere are then computed depending on FLI. SIMFIRE-BLAZE is incorporated within the LPJ-GUESS dynamic vegetation model^62,63^

### LPJ-LMfire fire model description

The LPJ-LMfire model^29,64^ includes processes to simulate natural wildfires from lightning ignition and a unique representation of fire generated by both agricultural and pastoral societies. To run LMfire we combined climatological mean climate fields that provide a high-spatial-resolution baseline, with detrended, interpolated climate anomalies from reanalysis simulations that provide interannual variability. The original datasets used to drive the model are described in Table 3 of Pfeiffer et al.^29^; here we describe the process that were used to prepare the interannually variable climate driver input files.

A 20th Century Reanalysis^65^ is used to impose interannual variability in our simulations in several steps. First, we prepared monthly anomalies relative to the period 1961–1990 for: mean temperature, diurnal temperature range, total precipitation, number of days with precipitation, cloud cover, wind speed, and convective-available potential energy (CAPE). These monthly anomalies are then detrended using a robust loess regression filter^66^ with a bandwidth of 30 years. Next, we interpolated the detrended anomalies for each meteorological variable to 0.5° spatial resolution. Next, a climate driver dataset was generated from a 1080-year climate dataset by dividing the 140-year detrended climate anomalies into twelve 30-year blocks at 10-year intervals. Anomalies were then sampled using a pseudo-random scheme where once a block was chosen it was not selected again until all of the other remaining blocks have been selected. In order to generate a 1080-year timeseries 36 blocks were selected, i.e., each block was used three times but appeared in a different order each time. Finally, each anomaly timeseries was added to its complementary baseline climatological mean climate field, and used the resulting 1080-year (12960-month) climate data input file to run LMfire. For the PI simulation in LMfire, we used only the last 150 years of this model run, i.e., years 931–1080, as this is the period where all of the model pools are considered to be in equilibrium, although we acknowledge that the actual state of the terrestrial biosphere in the late preindustrial Holocene may have been far from equilibrium in many regions^33,67,68^. Atmospheric CO_2_ concentrations were set to 1770 levels for the duration of this simulation.

In the LMfire PI simulation, land use is prescribed from the KK10 scenario^67^ for the year 1770. The managed burning routine of the fire module^29^ defines that 50% of the litter on 20% of the used land (cropland and pasture) is burned annually. As the seasonality of burning on used land is not well known at global scale, fire emissions are partitioned evenly across all snow-free days of the year with mean temperature above 0 °C. In reality, most biomass burning on used land probably occurred during certain periods of the year, e.g., after harvest, but it is currently beyond the capability of any global land use scenario to prescribe these periods.

To perform a PD simulation with LMfire, we made a transient model simulation for the period 1701–2000 driven by the synthetic climate timeseries described above, transient land use, and reconstructed and measured atmospheric CO_2_ concentrations. Land use was provided by merging KK10, which ends in 1850, to the Hundred Year Database for Integrated Environmental Assessments (HYDE) dataset following the protocol described in Kaplan et al.^68^. The LMfire PD results presented here represent the mean over the period 1991–2000.

Modern agricultural practices and legislation to protect air quality mean that in some parts of the world intentional burning of used land is currently rare. To account for this in the PD simulation, we set the burning-on-used-land factor described above to not exceed the observed burned area fraction in the JR12 burned area dataset^29,69^, which leads to burning on used land at PD to be effectively zero in large parts of North America and Europe.

Although passive fire suppression as a result of land use is included in this simulation following Pfeiffer et al.^29^, and burning on used land is largely eliminated in many parts of the world, the PD simulation of LMfire still overestimates mean annual burned area in parts of the world, notably in the western United States, southern Europe, and the Middle East. Because these are PD regions known to be subject to large-scale industrial fire suppression efforts, we accounted for this fire suppression by further correcting the LMfire output in a model postprocessing step. To identify regions affected by potential model bias, we compared modelled burned area with GFED4s at the level of GFED regions, with Europe further subdivided into North and South at 45° N (Supplementary Figure 9). Within those regions where the discrepancy between modelled and observed burned area was ≥90%, i.e., the USA, southern Europe, and Middle East, we assumed that the primary reason for the discrepancy was active fire suppression. To correct this model PD bias in these regions, we scaled the modelled BC emissions by the ratio of GFED4s observed to LMfire modelled burned area calculated at the GFED regional level. This resulted in a slightly <10% decrease to the global BC annual emission flux. In all other regions, we used modelled BC emissions without bias correction because the discrepancy was smaller and we could not justify industrial fire suppression as being the potential primary cause of model-data mismatch. The primary region where simulated burned area remains overestimated by LMfire is in the tropical seasonal forests of South America. This is both a region of rapid land use change over recent decades where anthropogenic influences on burning may not be well captured in the PD LMfire simulation, and where it has already been identified^29^ as a place where the process representation of fire could be improved in the LMfire model.

In all three GFED regions (encompassing the USA and Mediterranean) where PD LMfire emissions were scaled, the scaling reflects a modern-day fire suppression practice driven largely by technologies and ideologies that were not present in 1750. For example, in the PD US > 99.5% fires are actively suppressed^32^, and due to this the USA underwent reductions in annual burned area over the 20th Century of over 80% from >40 m ha in late 1920s to ≤5 m ha in the early 2000s (http://www.fao.org/docrep/010/ai412e/AI412E06.htm)^70^. We therefore assume that a similar small (<10% global emission reduction) PI bias correction is not required here. This does however highlight once more that fire models should aim to move beyond present-day optimisations^27^ by incorporating more paleofire data.

Differences in fire emissions within boreal regions between LMfire and GFED4s are in part due to the longer simulation time within LMfire (150 years) compared to satellite measurements, resulting in fires being simulated in the model that are not represented in the satellite record used to construct the GFED4s emission dataset. In particular, the wide range of fire regimes within the Arctic tundra leads to fire return periods of between decades to millennia^71^. Many high latitude fires are therefore unaccounted for in fire emission datasets^72^, including the CMIP6 and SIMFIRE-BLAZE datasets presented here, but can occur within LMfire. Other differences include assumptions about fuel load, combustion completeness, emission factors, permafrost, PD fire suppression and increased post-harvest agricultural burning in the PI that is not present in the PD due to land use change practices. Within semi-arid regions LMfire predicts a shorter fire return interval^29^ than suggested by recent studies^73^, however total emissions from these regions are generally low, and therefore less important for climate, due to the sparse vegetation cover within them^29^.

To test the sensitivity of PI fire emission estimates to natural climate variability the 150-year emission dataset of LMfire was partitioned into 15 decadal mean fire climatologies, each representing a plausible PI fire landscape. As tropical and extra-tropical fires belong to distinct regimes with different characteristics, four different scenarios (Supplementary Table 2) based on the maximum and minimum emissions from each were selected. These four scenarios were then used to generate the uncertainty range in estimates of LMfire emissions in Fig. 1 and the range in radiative forcing calculations in Supplementary Table 2.

### Modelled fire emissions

Dry biomass burned from both fire models is used to calculate fire emissions (mass of aerosol species emitted per mass of dry matter burned) in each grid cell using carbonaceous aerosol emission factors from Li et al.^74^ for non-herbaceous vegetation and emission factors from van der Werf et al.^31^ for herbaceous vegetation and sulphur dioxide emissions.

Total biomass available as fuel (Supplementary Figure 3) is calculated for SIMFIRE-BLAZE as the sum of carbon in living vegetation and the carbon in litter, and for LMfire as the sum of living vegetation in herbaceous vegetation and the carbon in litter. The spatial distribution of PI biomass is largely similar to the PD^75^ with peak biomass amounts simulated in SH tropical and NH boreal regions and appreciable biomass amounts in NH mid-latitudes (particularly around the Mediterranean and Eastern USA and China regions). Burned area (Supplementary Figure 3) is defined as the annual mean fraction of the grid cell which burns over the studied simulation time frame. Burned area in LMfire is calculated based on the well-established Rothermel equations of rate of fire spread, so fires spread only when fuel is available. Globally, modelled maxima in burned area for both LMfire and SIMFIRE-BLAZE do not exceed PD maxima observed burned areas within African Savannah regions^73^.

Different representations of anthropogenic land cover and land use change over the industrial period in each fire dataset contribute significantly to the difference in PI emissions at northern mid-latitudes (Fig. 1). The AeroCom and CMIP6 datasets assume that anthropogenic land cover does not change much over time, which is unrealistic^76^, and therefore PI emissions are spatially co-located with observed PD emissions. The SIMFIRE-BLAZE simulation incorporates the HYDE 3.1 dataset^77^ within the host LPJ-GUESS vegetation model, which linearly extrapolates historical land cover change based on 1961 land use, and results in increases to anthropogenic land use in western Europe at 1750^38,39,67^. The LMfire simulation incorporates the KK10 dataset^67^ within the host LPJ vegetation model, which assumes a Boserupian view of non-linear land use intensification where low population densities lead to fast anthropogenic land expansion^39,67^. The LMfire simulation therefore features significantly more anthropogenic land use in the early PI period than SIMFIRE-BLAZE, particularly over Eurasia, India, southeast Asia and Africa^39,67^. As LMfire incorporates a dedicated modelling scheme for simulating post-harvest agricultural fires, the extra impact of agricultural fires on emissions from cropland areas are contributing to the larger emissions in these regions compared to SIMFIRE-BLAZE. In these simulations 10% of the annual agricultural biomass is assumed to be left on the field post-harvest and subsequently burned each year.

LMfire emissions are approximately a factor 2 higher than SIMFIRE-BLAZE while CMIP6 approximately a factor of 2 lower. This level of uncertainty in fire emissions is half that reported in Lee et al.^78^ and common place when comparing modelled fire-related aerosol optical depth (AOD) to measured AOD. For example, Reddington et al.^79^ found that discrepancies between modelled aerosol and measured AOD and surface PM_2.5_ from biomass burning can be resolved by scaling fire emissions by a factor of 1.5–3.4, depending on dataset. They also note that other models require a scaling as high as a factor of 6, although 2–3 is more common. We therefore suggest that our uncertainty (factor of 2) in PI emissions reflects this current PD uncertainty—but is not higher and hence not unrealistic.

### Global aerosol microphysics model description

The GLObal Model of Aerosol Processes (GLOMAP) was used to calculate monthly mean aerosol mass and number concentrations in seven lognormal modes (one soluble nucleation mode, plus one insoluble and one soluble for each of the Aitken, accumulation and coarse modes)^80^. This study follows the experimental set up of Hamilton et al.^30^ with the addition of CMIP6 fire emissions for the PD. GLOMAP simulates particle nucleation, growth, coagulation, cloud cycling and deposition. The horizontal grid resolution is 2.8° × 2.8° with 31 vertical levels between the surface and 10 hPa. Modelled aerosol transport in both time periods is prescribed by 3D gridded wind speed, temperature and humidity fields for year 2008 from the European Centre for Medium-range Weather Forecasts (ECMWF), which are interpolated every 6 h. Cloud condensation nuclei (CCN) number concentrations are calculated at 0.2% supersaturation following Petters and Kreidenweis^81^, we assign hygroscopicities (*κ*) values of 0.61 for sulphate, 1.28 for sea salt, 0.1 for organic carbon and 0.0 for black carbon (BC). Aged soluble aerosols in GLOMAP are internally mixed and contain the species: sulphate, sea salt, organic carbon, BC and dust. We report CCN concentrations at the 915 hPa model level (~850 m a.s.l.), approximately corresponding to cloud base for low-level warm stratiform clouds. GLOMAP-mode has previously been used to model aerosol number concentrations in various studies involving biomass burning aerosols^30,82–85^ and has been shown to perform well against CCN measurements in different environments^86^. In the PI fossil fuel emissions and the concentration of anthropogenic volatile organic compounds are assumed to be zero in all scenarios. A small PI biofuel component exists, mainly due to domestic heating and cooking^14^. Natural emission fluxes of ocean dimethyl sulphide^87^, sea spray and dust to the atmosphere are generated as a function of the local wind speed^88,89^, resulting in identical emission fluxes for each time period. Biogenic volatile organic compound^90^ and volcanic emissions^91^ are also identical in both time periods.

### Ice core black carbon and fire tracer comparison

The Wyoming ice core^12^ is located at 43.1° N, 109.6° W and 4100 m above sea level. The D4 Greenland ice core^10^ is located at 71.4° N, 44.0° W and 2713 m above sea level. The North Greenland Eemian (NEEM) ice cores^42^ at 77.5° N, 51.2° W and 2480 m above sea level. The Colle Gnifetti (CG) Swiss-Italian Alps ice core^44^ at 45.6° N, 7.5° E and 4450 m above sea level. Each site has BC measurements for different historical periods: D4 1788 to 2002; NEEM 78 to 1998; CG has a single year PI measurement at 1750 and an average PD measurement between 1950 and 1980. The CG site is located within a heterogeneous alpine environment and BC concentrations in both time periods are calculated as the average concentrations from the model level matching the ice core site over six grid cells: the grid cell containing the CG site plus the grid cells directly to the east, west, south, southeast and southwest, those adjacent to the north were not included as it is assumed the Alps would provide a barrier to continental air flow. As the Greenland sites are within a more homogenous Arctic environment BC concentrations in both time periods are calculated at the ground level within the closest four grid cell containing the site.

While our model does not directly calculate BC concentrations in snow, as the processes involved are not represented, deposition data was retrieved from the model in a previous study^37^ and tested offline within the Community Land Model, which does represent the relevant processes. Results from this study showed that GLOMAP simulates BC deposition close to the mean for the Arctic region^37^. We therefore expect relative BC changes in air and in ice to be approximately comparable. Ice core measurements of fire emission tracers are sparse, limiting our analysis to BC, levoglucosan and vanillic acid (Supplementary Figure 1). We therefore assume that as the trends in these three species are generally similar pre-1880 (wide scale industrialisation) that POM and SO_2_ will follow similar trends. Limitations in comparing PI ice core measurements to PI modelled atmospheric concentrations centre on a lack of understanding of how the PI atmospheric state was behaving^59^. For example, there are uncertainties in aerosol transport and residence time, chemical transformation and deposition processes to Arctic regions^92^. Furthermore, in this study we assume that these processes are the same in the PI as the PD, although some could be different^93^, and by using a single year’s meteorology we do not capture possible changes to modelled deposition process (e.g., precipitation rates) at the ice core location. Another issue is relating the modelled regional aerosol emissions and processes to the point measurements of a single ice core, which can also capture emissions from varying sources. To account for uncertainties in the PI period caused by natural variability in fire activity and atmospheric circulation, the Greenland and Wyoming ice core BC concentrations in the PI were calculated as a 20-year mean (D4: 1788–1807, NEEM: 1740–1759, Wyoming: 1747–1766). In the PD Greenland ice core BC concentrations are also heavily influenced by industrial emissions that have been decreasing in strength over the past decades. We therefore calculate Greenland ice core means over a shorter 5-year period (D4: 1998–2002, NEEM: 1994–1998), and the Wyoming ice core over a 10 year period (1970–1979). The PD/PI ratio range was calculated using the standard error of the mean. The mean PD/PI BC, ratio for 5, 10 and 20-year averages in both time periods, and the standard errors, are shown in Supplementary Table 1. Mean PI BC concentrations from the Wyoming ice core are highly sensitive to the chosen PI start year; altering from 1747 (first year in the record) to 1750 (first year in the fire emission datasets) would increase mean PI BC concentrations by 23%, further reducing the measurement-based PD/PI BC ratio. For model M and observation O the symmetric bias factor was calculated as: For M > O: (M/O)−1; For O > M: –(O/M) + 1. Uncertainty in the CMIP6 industrial BC emission inventory used to calculate the decrease in BC emissions from anthropogenic activity in the region of the WY (factor of 1.6) and CG (factor of 2) glacier ice cores could be up to a factor of two^78^, however as the uncertainties in European and American BC emission datasets are much lower than other regions^43^ we estimate the uncertainty at ±25%.

Specific fire emission tracers are also recorded within the Greenland ice cores: yearly measurements of Vanillic acid (VA) at D4 and sub-decadal (~2 measurements decade^−1^) Levoglucosan (LG) measurements at NEEM. The mean PD/PI VA and LG ratio for 5, 10 and 20-year averages in both time periods, and the standard errors, are shown in Supplementary Table 1. The sub-decadal NEEM data has a large fire year within the 20-year mean (155 ng/g LG compared to a mean of 44 ng/g LG without that year) and therefore the PD/PI ratio is shown both with and without this year included. Measurements of LG and VA show that PD fire emissions deposited to Greenland have decreased since the PI, further supporting the fire model emission data (Supplementary Table 2).

### Charcoal database comparison

The Global Charcoal Database (GCD version 3.0.3) was used within the paleofire R package (http://cran.r-project.org/web/packages/paleofire/paleofire.pdf) to determine trends in fire activity over the industrial period (1750–2000). The GCD contains 736 charcoal records (NH: 436, SH: 300). Following Marlon et al.^9^ charcoal accumulation records in the GCD are transformed in a 3 step process prior to examination to allow comparison of records that are based on a wide range of sampling techniques. Step 1: minimax transformation to rescale values. Step 2: Box-Cox transformation to homogenise the variance within individual time series. Step 3: rescaling values to Z-scores. A LOcally WEighted regression Scatter plot Smoother (LOWESS) smoothing of the data was employed with a 10-year window half-width (i.e., a 20 year smoothing). Bootstrap analysis (1000 samples by site) gives a 97.5% confidence interval. The main difference in the charcoal analysis presented here compared to those in previous studies^8,9,94^ is that the charcoal influx anomaly base period for all panels is 1750–2015 CE. This time period is omitted from the base period in other charcoal reconstructions so as to avoid the impacts of anthropogenic activity on the results, which is what we wish to capture in this study.

### Aerosol radiative forcing calculation

We calculate the aerosol direct radiative forcing and cloud albedo forcing, also known as the first indirect forcing, between the PI and PD using the Suite Of Community RAdiative Transfer codes based on Edwards and Slingo (SOCRATES)^95^, with nine bands in the longwave (LW) and six bands in the shortwave (SW). We use an offline configuration driven by year 2000 monthly mean temperature and water vapour concentrations from ECMWF reanalysis data. We use a monthly mean cloud climatology (1983–2008) from the ISCCP-D2 archive^96^. The same PD simulation (with CMIP6 fire emissions) was paired with the three PI simulations using CMIP6, SIMFIRE-BLAZE and LMfire fire emissions. Both PI and PD fire emissions are used to calculate the AeroCom radiative forcing values in Supplementary Table 2 in order to provide a reference to compare the radiative forcing values calculated by using the CMIP6 PI and PD fire emissions. Each paired PI–PD simulation accounts for changes in anthropogenic aerosol emissions from 1750 to 2000 (due to industrial, transport and domestic fossil fuel combustion sources)^14^.

The direct radiative forcing is calculated as the difference in the net (SW + LW) top-of-atmosphere all-sky radiative flux between the PI and PD, based on the aerosol optical properties (scattering and absorption coefficients and the asymmetry parameter) for each size mode and spectral band^97^. The cloud albedo forcing is determined from the radiative perturbation induced by the change to cloud droplet number concentration (CDNC) between the PI and PD^48,98^.

Cloud droplet number concentrations are calculated^99–101^ from the monthly mean aerosol size distribution, assuming a uniform updraught velocity of 0.15 m s^−1^ over ocean and 0.3 m s^−1^ over land. The critical supersaturation is calculated using the hygroscopicity parameter (*κ*) approach^81^. A multi-component *κ* is obtained by weighting individual *κ* values by the volume fraction of each component. We assign *κ* values as follows: sulphate (0.61, assuming ammonium sulphate), sea-salt (1.28), black carbon (0.0), and particulate organic matter (0.1).

To calculate the cloud albedo forcing, a uniform control cloud droplet effective radius (*r*_e1_) of 10 µm is assumed to maintain consistency with the ISCCP derivation of the liquid water path. For each paired PI−PD experiment the effective radius (*r*_e2_) for low- and mid-level clouds (up to 600 hPa) is calculated as in Eqn. 1, from the monthly mean cloud droplet number fields CDNC_1_ and CDNC_2_, respectively (where CDNC_1_ represents the PD simulation, and CDNC_2_ represents the PI simulation).

1 $${r}_{{\mathrm{e}}2} = {r}_{{\mathrm{e}}1} \times \left[ {\frac{{{\mathrm{CDNC}}_1}}{{{\mathrm{CDNC}}_2}}} \right]^{\frac{1}{3}}$$

In these offline experiments, we do not calculate the second aerosol indirect (cloud lifetime) effect.

### Code availability

The codes used to conduct the analysis presented in this paper can be obtained by contacting the corresponding author (D.S.H.).

### Data availability

Data are available upon request from the corresponding author (D.S.H.).

## Electronic supplementary material

Supplementary Information
