# Supplementary material for: Reassessment of pre-industrial fire emissions strongly affects anthropogenic aerosol forcing
Source: Nat Commun. 2018 Aug 9;9:3182. doi: 10.1038/s41467-018-05592-9 (PMC6085333; doi:10.1038/s41467-018-05592-9)
Supplement: Supplementary file 1 — Supplementary Information [file 41467_2018_5592_MOESM1_ESM.pdf]

Reassessment of pre-industrial fire emissions strongly affects anthropogenic aerosol forcing.

Hamilton et al.

Supplementary Information.

Supplementary Table 1. The PD/PI change in black carbon (BC) and specific fire trace gases (Vanillic Acid (VA) at D4 and Levoglucosan (LG) at NEEM) concentrations with standard error of the mean (SEM) at two Greenland ice core sites (D4 and NEEM) calculated using different averaging time periods. NEEM value in parentheses includes a recent extreme high measurement.

	5 year PD 5 year PI		10 year PD 10 year PI		20 year PD 20 year PI		5 year PD 20 year PI	
	D4	NEEM	D4	NEEM	D4	NEEM	D4	NEEM
BC PD/PI	1.44	0.91	1.01	1.72	1.06	1.64	0.71	1.20
BC SEM	0.30	0.33	0.37	0.29	0.22	0.13	0.15	0.21
VA PD/PI	1.32	-	0.73	-	0.61	-	-	-
VA SEM	0.28	-	0.81	-	0.79	-	-	-
LG PD/PI	-	0.65	-	0.76	-	0.92(1.38)	-	-
LG SEM	-	0.12	-	0.14	-	0.40(0.28)	-	-

Supplementary Table 2: Fire aerosol and gas emissions for each of the four main PI experiment datasets, four PI climate sensitivity scenarios and single PD dataset used in the calculation of the aerosol radiative forcing between the PI and PD. The different representations of anthropogenic land use and modelled land cover behind each scenario are listed

						PI-to-PD	
Scenario	BC/ Tga^{-1}	POM/ Tga^{-1}	SO_2/ Tga^{-1}	Land use	Natural vegetation	CAF/ Wm^{-2}	DRF/ Wm^{-2}
PI AeroCom	1.03	12.80	2.92	PD scaled	PD	-1.34*	-0.71*
PI CMIP6 mean	1.61	18.87	2.00	Similar PD	GFED-Clim	-1.12	-0.68
PI LMfire mean	6.34	85.20	8.04	KK10	LPJ	-0.10	-0.62
PI Tropics Max	*6.20*	*80.49*	*7.81*	“	“	*-0.17*	*-0.62*
PI Tropics Min	*6.21*	*84.50*	*7.90*	“	“	*-0.12*	*-0.62*
PI xTropics Max	*6.75*	*90.73*	*8.53*	“	“	*-0.06*	*-0.62*
PI xTropics Min	*6.15*	*80.21*	*7.77*	“	“	*-0.17*	*-0.62*
PI SIMFIRE-BLAZE mean	3.30	46.36	4.25	HYDE 3.1	LPJ-GUESS	-0.73	-0.65
PD CMIP6	1.78	22.20	2.22	PD	PD		

$PI_{Aerocom}$= 1750, PI_{CMIP6}= 1750-1770, $PI_{SIMFIRE-BLAZE}$= 1750-1770, PI_{LMfire}= 1770 and PD= 2006-2015. BC=Black Carbon, POM=Particulate Organic Matter and POM= 1.4*organic carbon, SO_2=Sulphur dioxide. Tropics= 30°N to 30°S, xTropics= >30°N and >30°S. GFED-Clim= Global Fire Emissions Database climatology[1] based on Carnegie-Ames-Stanford Approach (CASA) biogeochemical model, KK10=Kaplan & Krumhardt 2010[2], HYDE 3.1=History Database of the Global Environment 3.1[3], LPJ=Lund-Potsdam-Jena dynamic global vegetation model[4–6]. CAF=Cloud Albedo Forcing and DRF=Direct Radiative Forcing. *Reference AeroCom PI-to-PD CAF and DRF calculated using AeroCom PI and PD fire emissions (see Methods).

Supplementary Table 3. Global and regional pre-industrial burnt area estimates. Regions defined as: Northern Hemisphere (NH), Southern Hemisphere (SH), United States (US), Europe (EU) and Australia (AUS).

Dataset	Pre-industrial global burnt area (Mha)	Burnt area percent contribution to global total				
		NH	SH	US*	EU**	AUS
LMfire	1180	60	40	6	4	14
SIMFIRE-BLAZE	640	43	57	3	2	8
Arora & Melton	630	61	39	5	2	5
GFED4s	660 [1]	* low LMfire agriculture emissions (~1%)				
FINN	1010 [2]	**high LMfire agriculture emissions (~37%)				

1. PD GFED4s burnt area (475 Mha) / PD to PI change in burnt area from Arora & Melton (0.72)
2. PD FINN burnt area (725 Mha) / PD to PI change in burnt area from Arora & Melton (0.72)

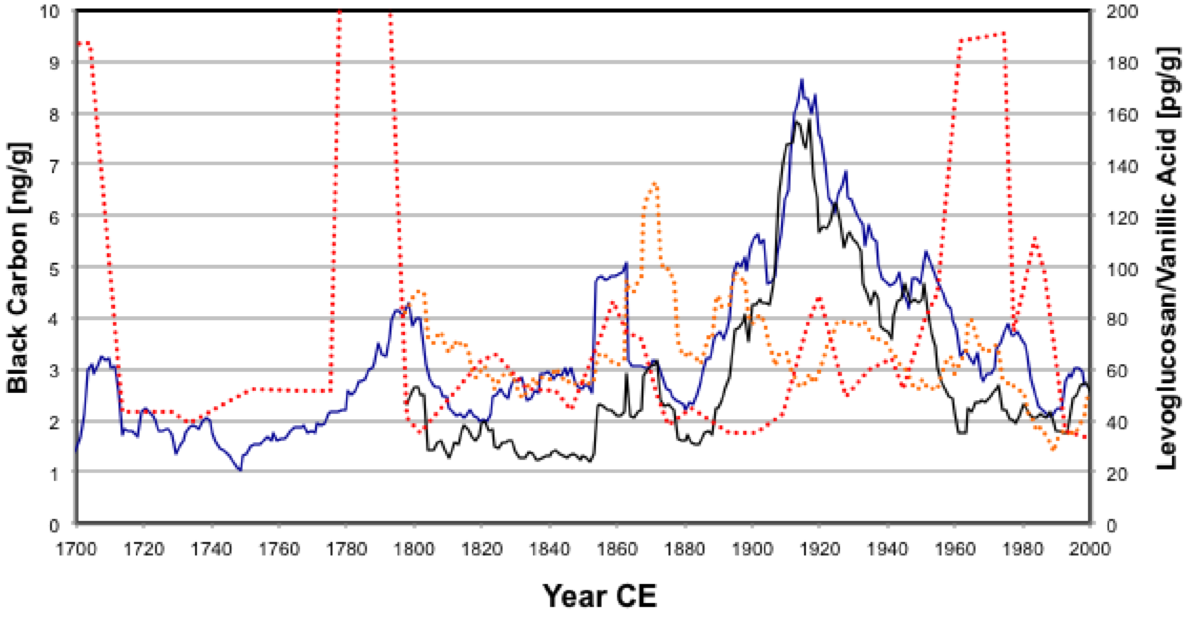

Supplementary Figure 1. Greenland ice core fire proxy concentrations. Measured change in black carbon (BC), levoglucosan (LG) and vanillic acid (VA) concentrations in two Greenland ice cores (D4 and NEEM) over the industrial period. Black line = 10 year moving average of annual BC measurements at D4. Blue line = 10 year moving average of annual BC measurements at NEEM. Orange dashed line = 10 year moving average of annual VA measurements at D4. Red dashed line = 2 point moving average of sub-decadal (~2 points/decade) measurements of LG at NEEM.

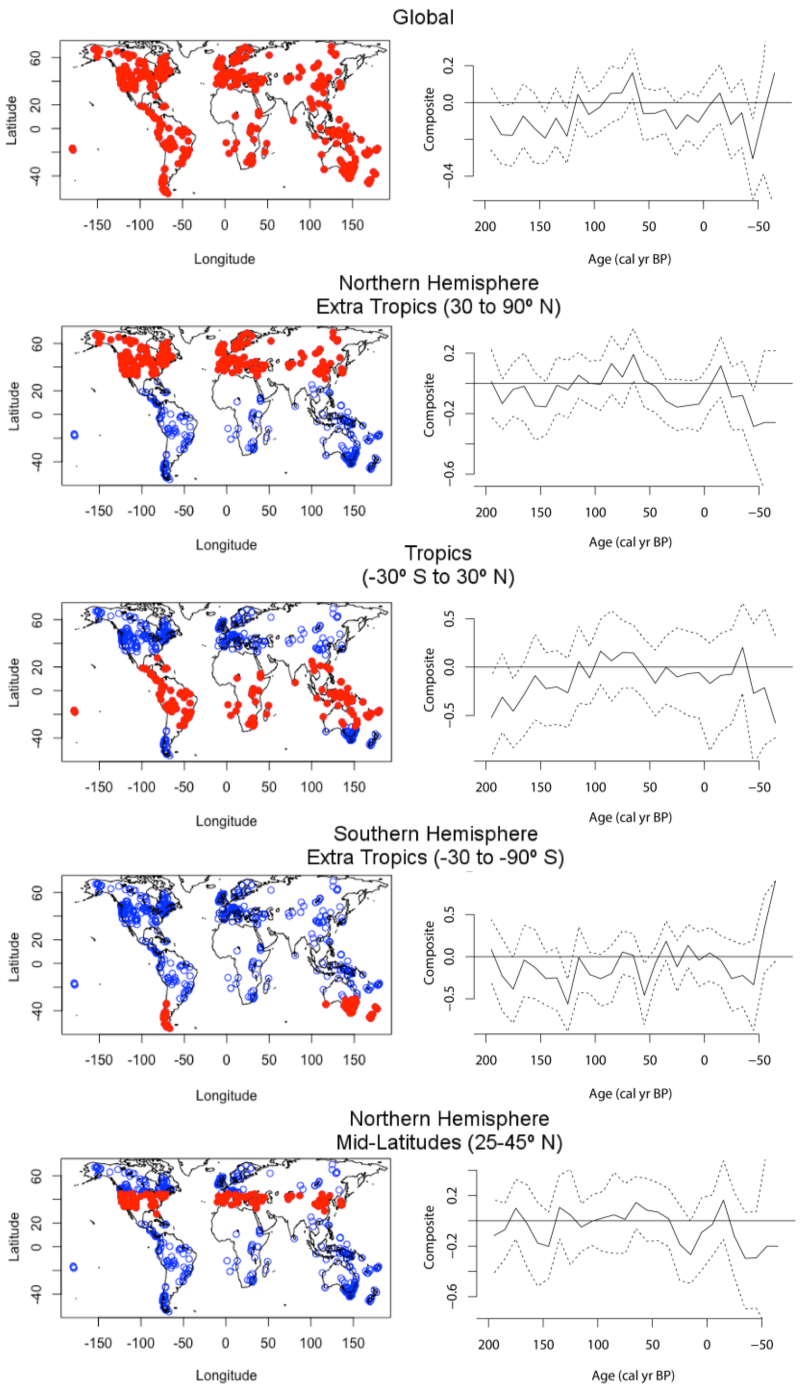

Supplementary Figure 2. Charcoal database analysis. Trends in biomass burning from charcoal composite z-scores (right hand panel) for five regions (left hand panel). The right column shows composites (red dots indicate locations in the corresponding left panel) calculated in a three-step transformation process (to allow comparison of different records): (1) minimax transformation of the charcoal accumulation record to rescale values; (2) Box-Cox transformation to homogenize the variance within time series; (3) rescaling values to Z-scores. In all left-hand panels circles show location of all charcoal samples. Charcoal influx anomaly base period for all panels is 1750-2015 CE. Bootstrap confidence intervals (97.5%) calculated from 1000 bootstrap samples are shown by dashed lines. Figures produced using `paleofire` R-package[7].

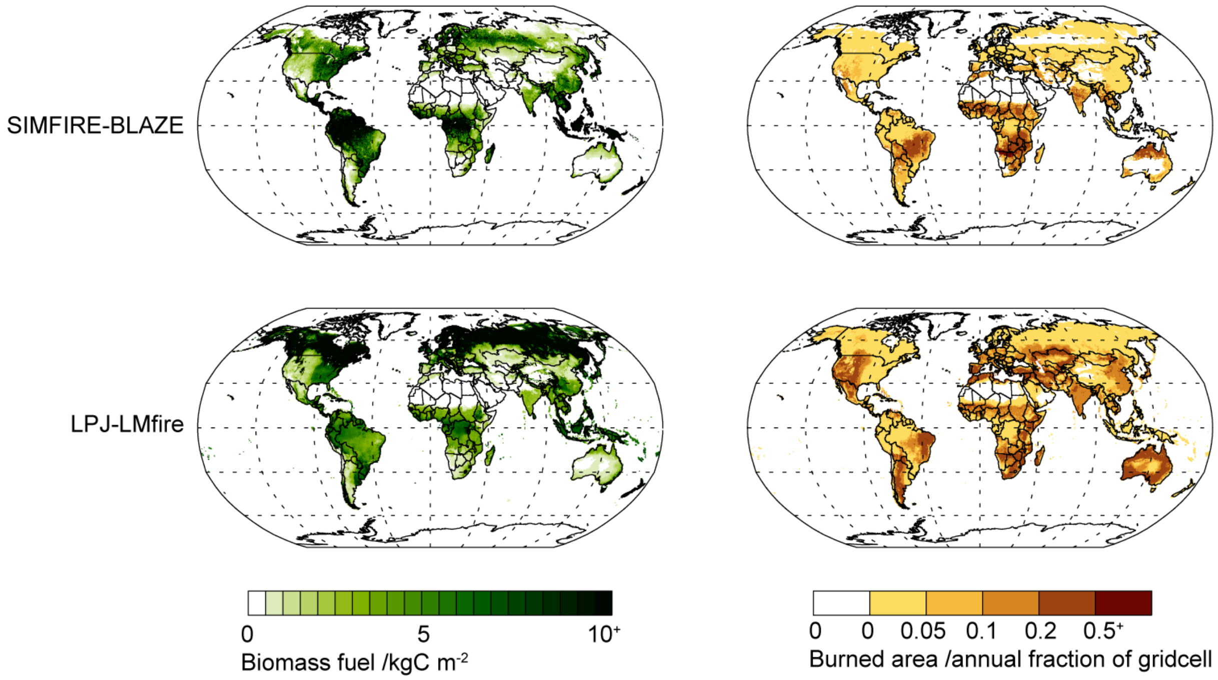

Supplementary Figure 3. Pre-industrial biomass fuel and burned area. Amount of pre-industrial biomass carbon available for fuel (kg/m2) and burned area (annual mean fraction of grid cell burnt) in LMfire and SIMFIRE-BLAZE. Averages taken over the same time-period as emissions.

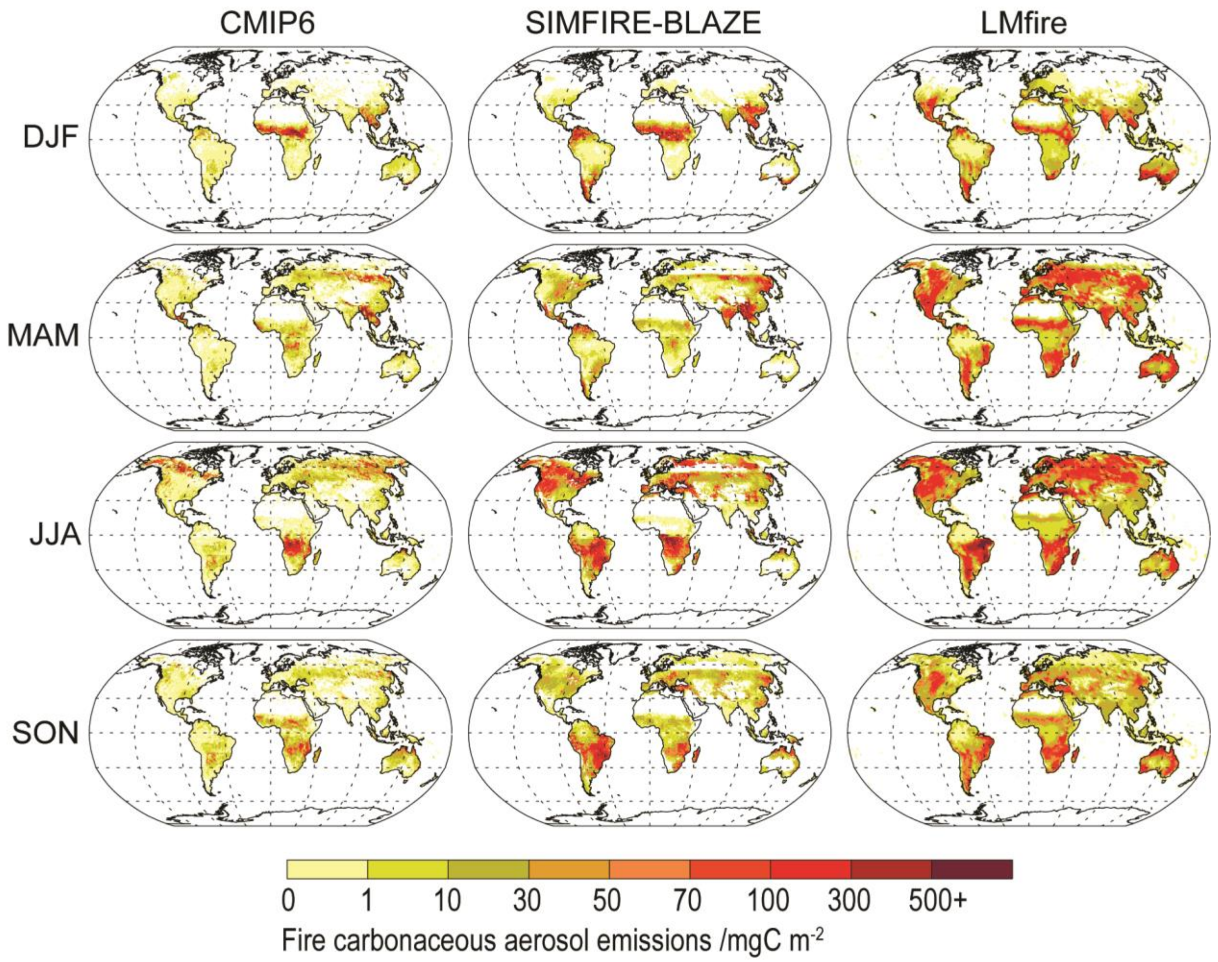

Supplementary Figure 4. Seasonal mean pre-industrial carbonaceous fire emissions. Pre-industrial (PI) emissions are sum of black and organic carbon for CMIP6 (PI: 1750-1770), SIMFIRE-BLAZE (PI: 1750-1770) and LMfire (PI: 1770) datasets. Winter: DJF (December, January, February). Spring: MAM (March, April, May). Summer: JJA (June, July, August). Autumn: SON (September, October, November).

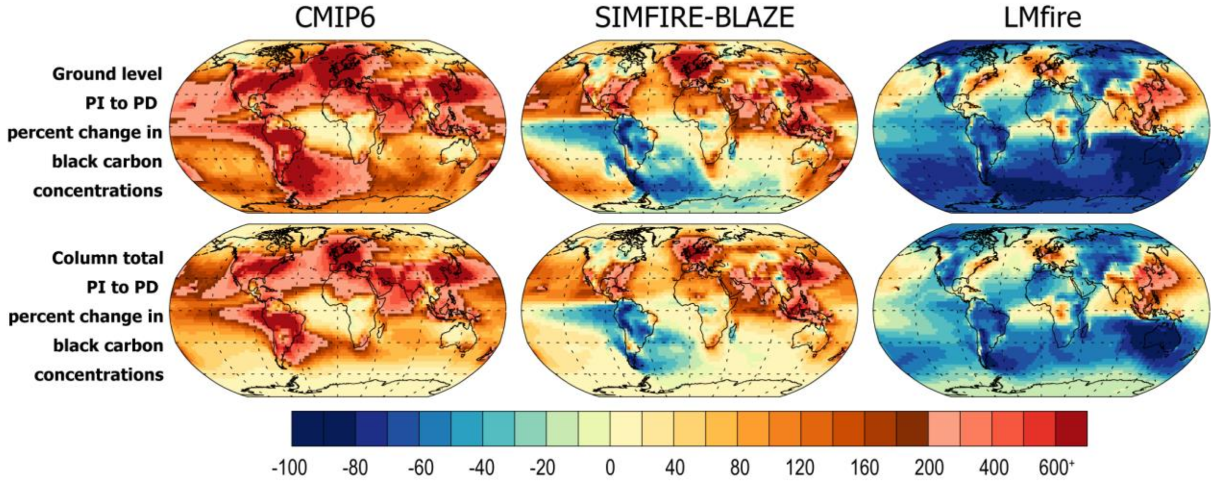

Supplementary Figure 5. Percentage change in modelled surface-level and column black carbon concentrations between the preindustrial (PI) and present day (PD).

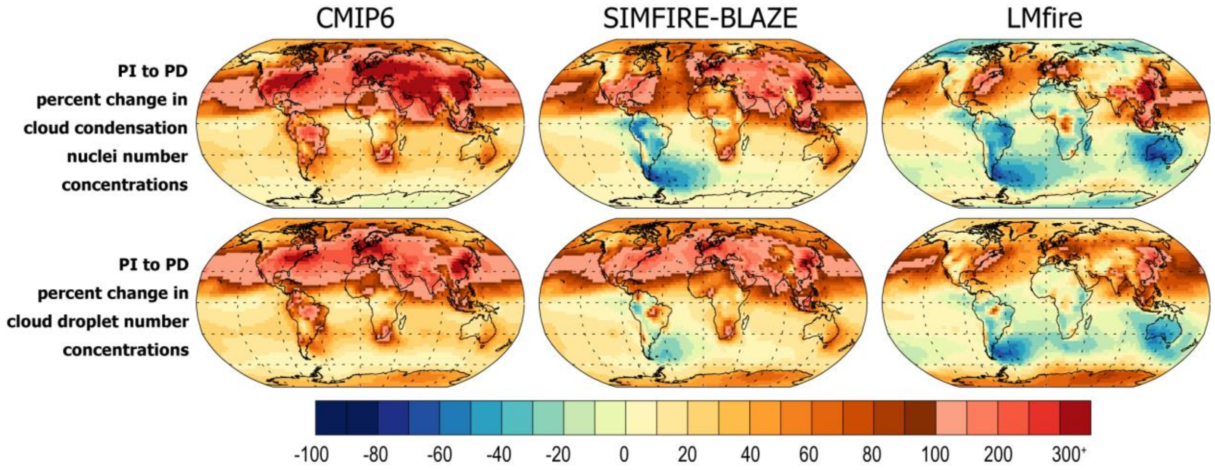

Supplementary Figure 6. Percentage change in modelled surface-level cloud condensation nuclei (CCN) and cloud droplet concentrations between the pre-industrial (PI) and present day (PD).

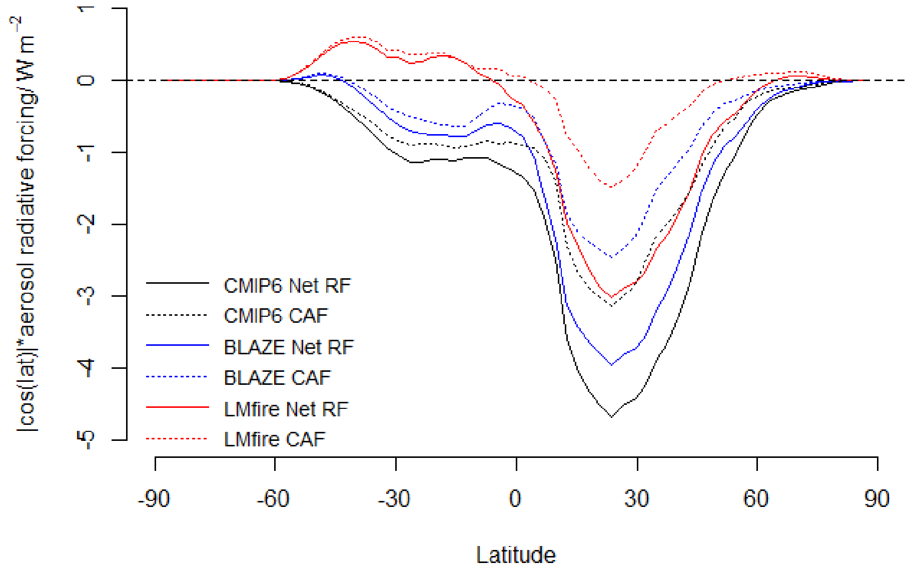

Supplementary Figure 7. Pre-industrial to present day annual mean cloud albedo forcing (CAF) and net aerosol forcing (CAF + direct aerosol forcing) as a function of latitude for the three simulations. Area weighted by multiplying by |cos(latitude)|.

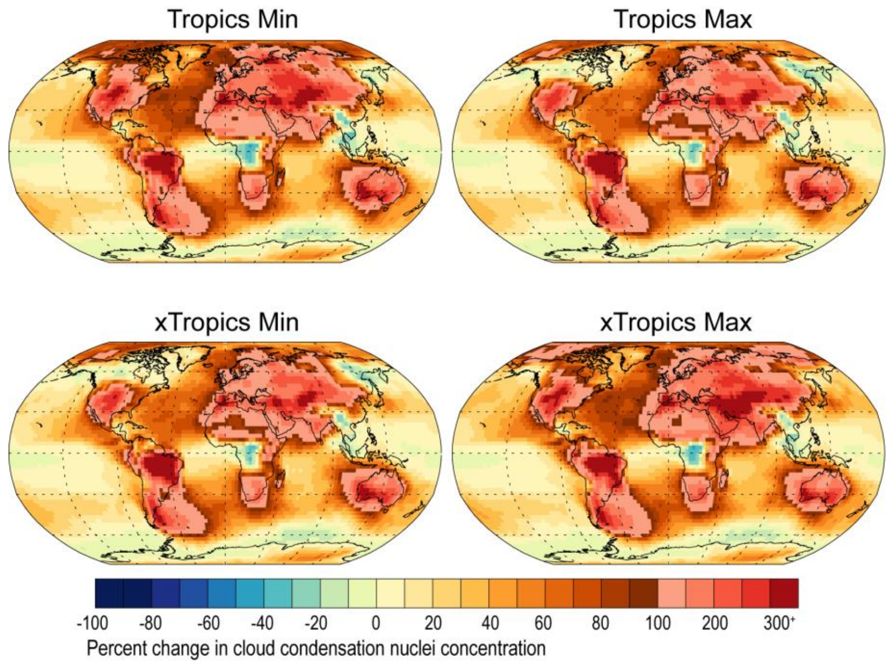

Supplementary Figure 8. Percentage difference between the modelled LMfire emissions and the CMIP6 1750 dataset for four natural variability LMfire scenarios: tropical and extra-tropical maximum and minimum in fire emissions. Each scenario is one of the 15 decadal mean fire climatologies, partitioned from the full 150 year LMfire dataset.

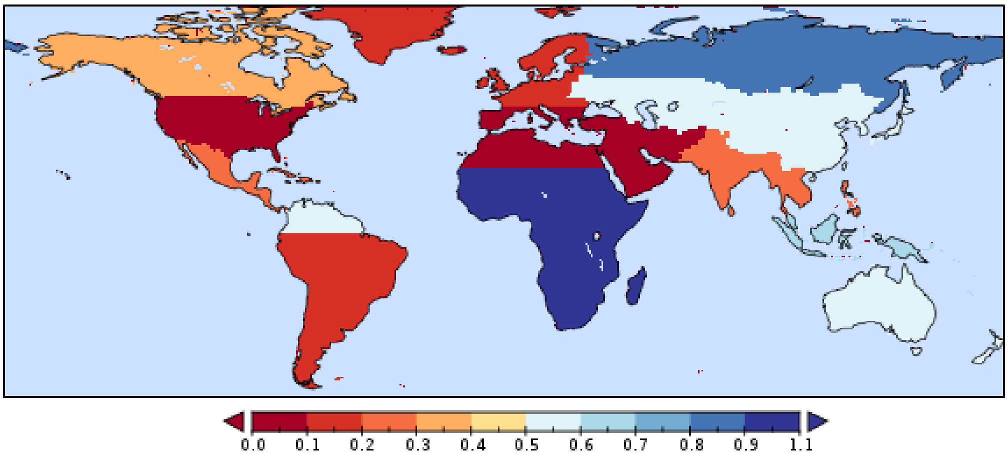

Supplementary Figure 9. Ratio of GFED4s burned area to LMfire burned area by GFED region.

References

1. Van Marle, M. J. E. *et al.* Historic global biomass burning emissions for CMIP6 (BB4CMIP) based on merging satellite observations with proxies and fire models (1750-2015). *Geosci. Model Dev.* **10,** 3329–3357 (2017).
2. Kaplan, J. O. *et al.* Holocene carbon emissions as a result of anthropogenic land cover change. *The Holocene* **21,** 775–791 (2010).
3. Klein Goldewijk, K., Beusen, A., Van Drecht, G. & De Vos, M. The HYDE 3.1 spatially explicit database of human-induced global land-use change over the past 12,000 years. *Glob. Ecol. Biogeogr.* **20,** 73–86 (2011).
4. Sitch, S. *et al.* Evaluation of ecosystem dynamics, plant geography and terrestrial carbon cycling in the LPJ dynamic global vegetation model. *Glob. Chang. Biol.* **9,** 161–185 (2003).
5. Smith, B., Prentice, I. C. & Sykes, M. T. Representation of vegetation dynamics in modelling of European ecosystems: comparison of two contrasting approaches. *Glob. Ecol. Biogeogr.* **10,** 621–637 (2001).
6. Smith, B. *et al.* Implications of incorporating N cycling and N limitations on primary production in an individual-based dynamic vegetation model. *Biogeosciences* **11,** 2027–2054 (2014).
7. Blarquez, O. *et al.* Paleofire: An R package to analyse sedimentary charcoal records from the Global Charcoal Database to reconstruct past biomass burning. *Comput. Geosci.* **72,** 255–261 (2014).
